# Comparisons of Intradialytic Exercise Versus Home-Based Exercise in Hemodialysis Patients: A Narrative Review

**DOI:** 10.3390/biomedicines12102364

**Published:** 2024-10-16

**Authors:** Chao-Lin Lee, Ping-Chen Wang, Yi-Ling Chen, Zen-Yong Chen, Ching-Cherng Uen, Hsien-Yung Lai, Chih-Chung Shiao

**Affiliations:** 1 Division of Neurology, Department of Internal Medicine, Camillian Saint Mary’s Hospital Luodong, No. 160, Zhongzheng S. Rd., Luodong 265, Yilan, Taiwan; chaolinlee@hotmail.com (C.-L.L.); danjinjong@gmail.com (Z.-Y.C.); venson208@yahoo.com.tw (C.-C.U.); 2 Department of Medical Research and Education, Camillian Saint Mary’s Hospital Luodong, No. 160, Zhongzheng S. Rd., Luodong 265, Yilan, Taiwan; pw279@oulook.com; 3 Department of Nursing, Camillian Saint Mary’s Hospital Luodong, No. 160, Zhongzheng S. Rd., Luodong 265, Yilan, Taiwan; olivia870815@gmail.com; 4 Department of Anesthesiology, Da Chien General Hospital, No. 36 Gongjing Rd., Miaoli 360012, Miaoli, Taiwan; 5 Division of Nephrology, Department of Internal Medicine, Camillian Saint Mary’s Hospital Luodong, No. 160, Zhongzheng S. Rd., Luodong 265, Yilan, Taiwan

**Keywords:** intradialytic exercise, home-based exercise, hemodialysis

## Abstract

With the increasing prevalence of end-stage kidney disease, the number of patients requiring hemodialysis (HD) continues to rise. While life-sustaining, HD is often associated with adverse effects such as muscle loss, physical deconditioning, fatigue, and compromised health-related quality of life (HRQoL). Recent research suggests that intradialytic exercise (IDE) and home-based exercise (HBE) may mitigate these adverse effects and improve patient outcomes. However, the existing literature mainly focuses on the outcomes of both exercises, whereas the comparison of types is often omitted. Hence, this review consolidates findings from studies investigating the effectiveness, implementation, safety, feasibility, and adherence of different types of IDE and HBE in HD patients. Overall, the current literature bolsters the significance of IDE and HBE for improving health in HD patients. IDE and HBE enhance physical function, cardiopulmonary capacity, HRQoL, and cognitive well-being. Some research proposed an indirect link between IDE and survival rates. Despite these benefits, challenges remain in implementing these exercise modalities, including patient adherence and the feasibility of routine exercise during HD sessions. Integrating these exercises into routine care allows healthcare providers to enhance outcomes for HD patients. Further research is suggested to optimize exercise protocols and explore long-term effects and cost-effectiveness.

## 1. Introduction

With advancements in medical technology, life expectancy has significantly increased. The aging society leads to a rise in the population of individuals with chronic kidney diseases, including end-stage kidney disease requiring hemodialysis (HD) treatment. Maintenance HD is the essential treatment for end-stage kidney disease (ESKD) patients, which often contributes to a series of adverse effects such as frailty, which consists of five main phenotypes, including unintentional body weight loss, self-reported exhaustion, weakness defined using muscle strength, slow walking speed, and low physical activity [1]. Compared to healthy individuals, HD patients easily feel exhausted and weak and have only one-fifth to one-half of the average activity rate [2]. This physical inactivity in HD patients keeps gradually worsening with a monthly declining rate of up to 3.4% [3], causing gradual muscle loss and weakness [3,4]. Consequently, patients often adopt the seated position, which further triggers a higher risk of mortality and morbidity [5], creating a vicious circle with a worse health-related quality of life (HRQoL) for HD patients. As widely known, a poorer physical component summary (PCS) of HRQoL is a crucial predictor of lower survival rates in HD patients. Physically inactive HD patients face a 62% higher risk of mortality within a year compared to their physically active counterparts [6,7], while the increased mortality risk is associated with reduced cardiovascular capacity, lower HRQoL, and a sedentary lifestyle [8].

Currently, two exercise modalities, intradialytic exercise (IDE) and home-based exercise (HBE), enhance HD patients’ health and well-being. These interventions hold the promise of improving HD patients’ overall health outcomes. IDE refers to physical activities during dialysis sessions, including cycling or resistance training. HBE is undertaken outside dialysis centers, including walking, stretching, and resistance exercises. Several studies have investigated the effects of various exercise types (e.g., aerobic, resistance, and stretching) in different settings (e.g., IDE and HBE) on various clinical outcomes. However, limited research has comprehensively compared the differences between different exercise types in clinical settings (IDE and HBE) regarding all the aspects of clinical utilization of these exercises. This review examines the benefits, safety, and adherence of IDE and HBE by evaluating the impact of different exercise regimens. We aim to provide insights into their potential benefits, safety considerations, and future directions for incorporating IDE into routine HD care. Our review seeks to contribute to the growing knowledge base on optimizing patient outcomes and enhancing HRQoL for HD patients.

## 2. Methodology for Literature Search

The methodology of this narrative review aimed to provide a comprehensive exploration and quick overview of the comparison between IDE and HBE. This approach allowed for the synthesization of diverse perspectives and findings across various studies. To address the limitations inherent in narrative reviews, we implemented several rigorous steps to maintain the research’s standard. This review searched studies in the PubMed and Google Scholar databases using the search terms ”intradialytic exercise,” “home-based exercise,” and “hemodialysis”. We selected clinical trials, review articles, and systemic reviews and meta-analyses published in English in the past decade (2014 to 2024). These studies were then analyzed to construct this narrative review.

## 3. Brief Overview of Effects of Various IDE and HBE Modalities

For IDEs, aerobic exercise is usually implemented through cycling for a minimum of 30 min and 2–3 times a week to increase breathing and heart rate [9,10,11]. Resistance exercise is accessed through weight machines, elastic bands, dumbbells, or repetition sets at least twice a week with low-to-moderate intensity to increase muscle endurance and strength [10]. Stretching exercise involves gentle stretching with a very low intensity to target specific muscle groups/and or joints, including quadriceps, gastrocnemius, and hamstrings stretching, soleus ankle plantar flexion, wrist extension, and rotational movement [12,13]. Additionally, some less commonly applied exercises include inspiratory muscle training for enhancing inspiratory muscle strength [14], neuromuscular electrical stimulation for creating an exercise mimetic to reach muscle contractions [15], and blood flow restriction exercise for decreasing arterial perfusion to the lower limbs [16].

As to the HBE, it is relatively form-bound-free. HBE can be generally categorized into aerobic (mostly home-walking), resistance, and combined exercise [4]. Due to the limitations of inadequate adherence, fatigue, or other reasons, home walking is predominantly applied in practice [4,17,18]. Home-based walking programs are typically tailored to patients’ functional abilities and involve daily sessions lasting 15 to 45 min [17]. These programs gradually increase in intensity over time. However, safety considerations related to geographic factors, patient conditions, comorbidities, and weather may lead to capping the exercise intensity [19]. Similarly, home-based resistance exercise is implemented through body weights, weight machines, or elastic bands with low-to-moderate intensity [4] (Table 1).

## 4. Merits of IDE and HBE

**Figure 1:**
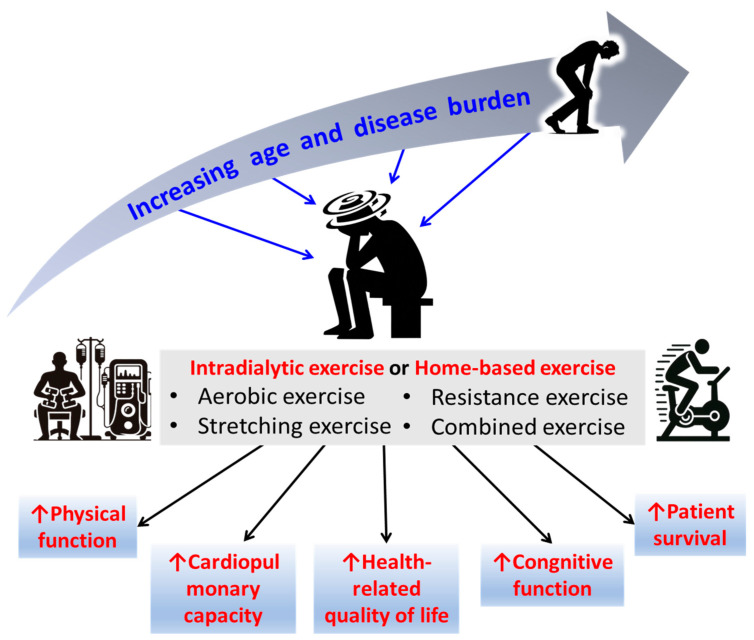
Summary of the types and benefits of intradialytic exercise and home-based exercise.

An increasing body of evidence supports the merits of IDE and HBE in preventing HD patients from being trapped in such a vicious circle brought by ESKD and HD treatment. Specifically, IDE has been shown to have benefits on the potential improvements in muscle loss, 6 Minute Walk Test (6MWT), sit-to-stand (STS) test, hand grip strength (HGS) test, cardiopulmonary capacity [9,28], patient survival rate [7], and HRQoL [8,29,30,31]. Likewise, HBE can benefit HD patients by improving walking capacity [17,32,33], muscle strength [4], physical functions [34], and cardiovascular capacity [21,32] (See Table 2 and Figure 1).

On the other hand, the benefits of HBE on HRQoL are still debatable. Myers found HBE can improve kidney disease quality of life (KDQoL) compared to the usual care group (*p* = 0.02) [24], while another research revealed no statistical significance regarding the improvement in mental component summary (MCS) (*p* = 0.57) and PCS (*p* = 0.12) [25]. Similarly, a meta-analysis including 12 studies found no significance in improving HRQoL [32].

### 4.1. Improving Physical Function

#### 4.1.1. Muscle Mass and Muscle Strength

Skeletal muscle mass in HD patients tends to decrease because of the adverse impact of ESKD and HD treatment [45]. This phenomenon is believed to result from less nutrition take-in, endocrine disorders, insufficient food intake, multiple endocrine disorders, enduring inflammation, etc. [46]. Research on IDE and HBE has highlighted the importance of preventing or regaining muscle loss and increasing muscle strength.

A randomized controlled trial (RCT) enrolling 44 HD patients (mean age 67.0 years, women 41%) found that aerobic IDE thrice weekly for seven months significantly maintained muscle thickness [36]. Another RCT, including 120 patients (mean age 48.9 years, women 44.2%), disclosed that aerobic and combined aerobic-resistance IDE were undertaken equally twice a week and significantly improved lower extremity strength [35]. These results were further confirmed by a meta-analysis including 14 RCTs (n = 837) demonstrating strong evidence for resistance IDE in enhancing muscle mass, strong to moderate evidence of resistance IDE, and moderate evidence for combined IDE for improving muscle strength [47]. On the other hand, home-based resistance exercises with moderate to high intensity increased muscle mass and strength successfully [48].

#### 4.1.2. 6MWT, STS Test, and HGS Test

An RCT enrolling 44 HD patients (mean age 67.0 years, women 41%) found that aerobic IDE thrice weekly for seven months significantly improved motor functions, including 6MWT, STS, and HGS [36]. A meta-analysis including 50 RCTs (n = 1757) found that, compared to the control group, 6MWT distances were significantly improved by aerobic IDE, resistance IDE, functional electrostimulation (FES), and IMT. IMT had the most pronounced effect (Effect sizes = 36.37 to 117.62 m, WMD = 117.62 m, 95% CI 67.26–167.99, I^2^ = 0%, *p* < 0.00001) [38]. Additionally, Zhang et al. conducted a meta-analysis to evaluate the effects of IDE on HD patients. In a subset meta-analysis including eight studies (n = 299), 6MWT was found to improve through resistance significantly IDE [standardized mean difference (SMD) = 0.517; 95% CI = 0.283–0.751, *p* < 0.001], while in another subset meta-analysis including five studies (n = 164), 30 s STS (STS30) was found to be significantly improved (SMD = 0.353; 95% CI = 0.123–0.583; *p* = 0.003) when compared to the control group, with no between-study heterogeneity [40].

These results were supported by a meta-analysis that disclosed that the IDE significantly increased HGS (from a subset of 7 RCTs with 254 patients, SMD = 0.58; *p* = 0.001) and 60 s STS (STS60) (from a subset of 7 RCTs with 425 patients, MD = 3.74; *p* < 0.001) [49]. Another meta-analysis, including 14 RCTs (n = 837), demonstrated moderate evidence for resistance IDE for improving 6MWT and short physical performance battery (SPPB), including STS and gait speed tests [47]. It is worth mentioning that a meta-analysis by March et al. enrolled RCTs that had reported sarcopenia-associated measurements and found that IDE increased HGS (in a subset of 7 RCTs with 127 HD patients; standardized mean difference, 0.58; *p* = 0.01) and STS60 (in a subset of 7 RCTs with 211 HD patients; mean difference, 3.74 repetitions; *p* < 0.001) [49].

As to HBE, a multicenter RCT by Manfredini et al. enrolled 296 HD patients (mean age of 63.5 years, women 34%) and randomized them to an exercise group undertaking home-based, low-intensity walking exercise, which was gradually intensified [17]. When compared to the control group, the 227 patients who completed the program experienced significant improvements in 6MWT (*p* < 0.001) and STS5 (*p* = 0.001) after a 6-month exercise [17]. These positive effects highlight the value of personalized HBE programs in enhancing functional status among HD patients [17]. A recent meta-analysis by Chen et al. enrolled 12 RCTs to evaluate HBE’s effects on ESKD patients. The results showed that long-term (3–6 months) HBE significantly enhanced patients’ 6MWT and VO2peak (*p* = 0.01) in ESKD patients. However, the HBE did not substantially affect HGS (*p* > 0.05) [32].

### 4.2. Improving Cardiopulmonary Capacity

Current literature has demonstrated that aerobic IDE might improve VO_2peak_ in ESKD patients. ESKD patients undergoing 2–6 months of aerobic IDE noticed a 17% increase in VO_2peak_ [9]; Pu et al. also support this finding (MD = 4.11, 95% CI = 2.94–5.27, *p* < 0.0001) [28]. However, the causal impact of VO_2peak_ and IDE is unclear, and the sample size might be controversial; hence, the results of IDE and HBE on VO_2peak_ might need further research to be consolidated. Likewise, Cheng et al. pointed out that adopting non-progressive or low-intensity IDE might not improve physiological functions or cardiovascular health [50].

On the other hand, several studies stand out for the significance of IDE and HBE on cardiovascular capacity. A meta-analysis including 50 RCT (n = 1757) disclosed that, compared to the control group, VO_2peak_ was significantly increased by aerobic and combined IDE (WMD = 2.07 and 5.41 mL/kg/min, respectively) [38]. Also, a meta-analysis including 50 RCT (n = 1757) disclosed that both aerobic and combined IDEs increased VO_2peak_ (Effect sizes = 2.07 to 5.41 mL/kg/min, WMD = 5.41 mL/kg/min, 95% CI = 4.03–6.79, I^2^ = 0%, *p* < 0.0001) significantly differ from the control group, of which combined IDE had the most pronounced effect [38]. A meta-analysis including seven studies further supports the stance found there was a significant difference in VO VO_2peak_ mL/kg/min value for aerobic exercise only in the fixed effect model (MD = −1.64 [CI = −3.21; −0.07]), with high and significant heterogeneity (I^2^ = 87%, R^2^ = 30.42, *p* < 0.01) [41].

Additionally, a nine-month hybrid IDE scheme recruiting 22 patients (mean age of 56.5 years, women 16.7%) conducted by Giannaki et al. found that long-term hybrid IDE could potentially lead to a rise in left ventricular ejection fraction, left ventricular function, and heart rate variability (HRV) parameter enhancement, indicating clinical significance for early intervention of cardiovascular events [23]. Cardiac afterload and augmented function of cardiac autonomic nervous system activity are expected to be reduced by such a hybrid IDE, as well as an increase in the oxygen supply of cardiac muscle [51]. Andrade et al. also proposed a similar perspective, concluding that IDE has benefited cardiopulmonary fitness, which further improves HD patients’ respiratory function and inspiratory/expiratory muscle strength [42].

Interestingly, prior research has pointed out that IDE could, to some extent, improve HRV parameters by increasing higher frequency (HF) and lowering lower frequency (LF) indices [23]. These HRV parameters have the potential to predict the incidence of major adverse cardiac or cerebrovascular events, which is one of the deadly urgencies for HD patients [52,53].

HBE can also improve VO_2peak_. A meta-analysis including seven studies found VO_2peak_ improved [32]; a meta-analysis including 24 studies revealed increased VO_2peak_ (MD = 4.11, 95%CI = 2.94 to 5.27, (*p* < 0.0001) [30]; VO_2peak_ improved through combined exercise compared to untrained patients [21]. However, a few studies argue that home-based walking was found to have no significance in improving cardiovascular capacity [17,18]. It is by far unsure which factor is causing such heterogeneity.

### 4.3. Improving HRQoL

#### 4.3.1. PCS and MCS

The introduction of IDE or HBE improved HRQoL, specifically the PCS of HRQoL. A 12-week research study of IDE intervention by Lin et al. revealed that HRQoL and depression in HD patients (mean age 62.1 years, women 35.9%) could be improved through IDE (Total scale of Cronbach’s alpha = 0.93 and 0.92) [29]. Recent research has also shown that applying aerobic and resistance IDE interventions could improve the PCS of HRQoL. [11,54]. Nevertheless, Salhab et al.’s research, incorporating 22 papers, concludes that aerobic IDE can improve mental and physical summary, whereas aerobic-resistance exercise only significantly improves PCS [8]. Similarly, Hu et al. revealed that most IDEs can improve PCS, yet aerobic IDE alone can reach the target [55].

Four RCTs concluded in a meta-analysis found that IDE could lower the depression level (SMD = −1.16, 95%CI −1.86 to –0.45) with no apparent heterogeneity (I^2^ = 77%, *p* = 0.01) [28]. PCS improved (MD = 7.72, 95% CI = 1.93 to 13.51, *p* = 0.01), with significant heterogeneity (I^2^ = 77%, *p* < 0.0001) [28]. MCS did not improve significantly (MD = 3.05, 95%CI = −1.47 to 7.57, *p* = 0.19) with no significant heterogeneity (I^2^ = 53%, *p* = 0.04) [28]. Further, a meta-analysis including five studies found that aerobic IDE can positively affect PCS and MCS. The included studies showed high heterogeneity [8]; a meta-analysis including 56 studies concluded that aerobic exercise alone has no significance in improving quality of life [43]; a meta-analysis including 56 studies found resistance IDE alone can improve PCS (9.53 points; 95% CI = −3.09, 22.15 points), compared with the control group [43]; a subset analysis from a meta-analysis, including 7 RCTs (n = 297), found no significant differences in PCS (SMD = 0.23; *p* = 0.055) and MCS (SMD = 0.13; *p* = 0.08) between resistance IDE and control group [40].

However, UK research recruiting 379 patients (mean age 59.4 years, women 37.6%) revealed that the IDE-induced changes in the PCS in 6 months only show a borderline significance (*p* = 0.055, Mean = 2.4, and 95% CI = −0.1 to 4.8) [31]. Similar results can be seen on other measures of HRQoL, implying an IDE intervention might not affect HRQoL [31]. However, it is believed that this result may be due to incompliance or inadherence, with only 47% of participants completing the IDE scheme and non-adherence, with only 18% of patients following exercise instructions [56]. It is reasonably believed that the results with higher participant compliance and adherence can echo the research results from previous studies. Further, the intensity might also affect the effectiveness of IDE and HBE. As bolstered by Takhreem et al., moderate-intensity exercise schemes significantly impacted HRQoL improvement [57]. Also, the IDE scheme was found to improve physical performance (MD = 61.81, 95% CI = 34.97 to 88.65, *p* < 0.0001), with no apparent heterogeneity (I^2^ = 0%, *p* = 0.78) [28].

On the other hand, current research has not agreed on the significance of home-based HRQoL; the discussion is still debatable. Myers found HBE can improve KDQoL compared to the usual care group (*p* = 0.02) [24], while another research revealed there is no statistical significance found on the improvement in MCS (*p* = 0.57) and PCS (*p* = 0.12), while RCS (*p* < 0.01) significantly improved [25]. Similarly, a meta-analysis including 12 studies found no significance in improving HRQoL [32].

#### 4.3.2. Fatigue Symptoms, Insomnia, Depression, and Muscle Cramps

Several studies emphasize IDE’s positive impact on HD patients’ fatigue symptoms [58,59]. Additionally, fatigue symptoms, IDE is also effective in improving sleep quality and mental well-being [28] and depression [60] among HD patients. A systematic review enrolling 15 studies found that muscle cramps can be mitigated by stretching IDE [61], while another study demonstrated the effect of stretching IDE on improving lower muscle cramps caused by fluid loss during intradialytic sessions [62].

On the other hand, HBE was also found to benefit depression in HD patients. A meta-analysis incorporating seven studies found that HBE can improve depression symptoms [63]. Similarly, Ortega-Pérez de Villar also confirmed the improved depression level after HBE intervention (*p*  =  0.02) [56].

### 4.4. Improving Cognitive Functions

Previous literature has agreed on the effectiveness of IDE in improving cognitive functions (SMD = 0.37, 95% CI = 0.13, 0.60, *p* = 0.002) [44]. Liu et al. also found this outcome significant under a minimum of 30 min IDE intervention and for HD patients under 65 years old (SMD  =  0.39, 95% CI = 0.10, 0.68, *p*  =  0.01) [44]. On the other hand, HBE does not significantly improve cognitive functions (*p* = 0.78) [33]. However, limited studies explore this field of outcomes; hence, the effectiveness might be controversial.

### 4.5. Improving Survival Rate

In a study examining the effect of a 6-month IDE intervention, researchers found that the intervention group (6%) had a lower mortality rate compared to the control group (27%) during the 12-month follow-up period (Log-rank statistics  =  6.5 and *p*  =  0.01). This suggests that IDE has clinical value in improving survival rates [7]. However, while limited research exists on the direct association between survival rates and IDE, several studies indirectly support the potential benefits of IDE on HD patients. For instance, Loon et al. investigated the relationship between HRQoL and survival rates, highlighting a positive association between physical functioning, emotional health, and social functioning with HD patients’ 2-year mortality rate [64]. Similarly, Tentori et al. demonstrated an association between various metabolic markers (such as mortality and parathyroid hormone) and abnormalities in serum calcium and phosphorus [65]. In addition, Isoyama et al. revealed that lower muscle mass and strength are independently correlated with higher mortality rates among HD patients [66].

These studies suggest that IDE indirectly impacts survival rates by influencing physical health, HRQoL, metabolic markers, muscle mass, and strength. However, further research is needed to establish a direct correlation between IDE and survival rates.

## 5. Comparisons in Clinical Benefits, Safety, Feasibility, and Adherence between IDE and HBE

**Figure 2:**
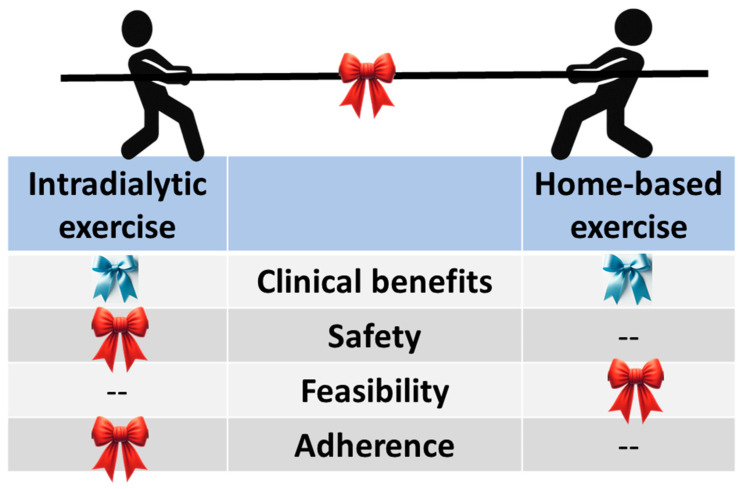
Diagram illustrating the comparisons between intradialytic exercise and home-based exercise. Note: blue and red bows denote “probably equal effect” and “probably better”, respectively.

Figure 2 illustrates the comparisons between IDE and HBE. With ongoing debates, current knowledge acknowledges that the clinical benefits of IDE and HBE are comparable [4,18]. As to the safety issues, the safety of IDE is supported by meta-analyses that indicated no increased risk for HD patients [28,54]. Some considerations regarding the safety of IDE, including intradialytic hypotension due to exercise-induced blood flow changes [67] and the risk of injury or falls, exist [28]. However, these risks can be minimized with appropriate monitoring, treatment adjustments, and tailored exercise programs [68]. On the other hand, as physical training is undertaken without supervision, HBE is potentially associated with higher safety concerns [69].

Regarding feasibility, IDE faces limited space, equipment, scheduling conflicts, and patient motivation [70]. Mitigation strategies include portable equipment, flexible scheduling, and patient education. HBE is more flexible, with no specific targets and fewer facility limitations, making it easier to execute but potentially lower adherence [4]. One obstacle to implementing exercise interventions is adherence. Although the adherence of IDE and HBE is still widely debated [4], it is generally accepted that IDE has a higher adherence rate (71% to 91% [71]) than HBE (53% to 80%) [72]. The core reason is the lack of supervision and flexibility in exercising intensity. However, despite the low adherence rate, research has found that exercise is effective [56] (Figure 2).

## 6. Discussion

### 6.1. Barriers to Implementation of Exercises

Although IDE and HBE benefit HD patients, introducing both exercises is controversial. High dropout rates [73], incompliance [31], and inadherence [4] make exercise intervention challenging if patients are unwilling or unable to follow the program. These issues are more prominent in home-based walking interventions due to the lack of supervision. Additionally, moderate-intensity IDE significantly influences participants [29,47], but all HD patients may not accept such intensity, creating further obstacles. The intensity and type of IDE must be tailored to individual capabilities and health conditions. Older participants or those with multiple comorbidities might struggle with higher-intensity exercises, requiring more staff for support or a personalized curriculum. This resource-intensive nature can challenge clinics, needing more equipment or staff for consistency. In contrast, HBE requires fewer facilities or equipment, with patients using available tools. However, this personalized approach may need more clinician assistance to tailor the program, and its effectiveness is more challenging to quantify and follow up on compared to clinic-based IDE.

### 6.2. Factors Potentially Influencing the Effects of Exercise

Several factors potentially influencing the impact of exercise in HD patients, which this review or the current knowledge cannot conclude, are worthy of discussion. The first discussed factor is adherence. As shown in the previous sections, higher-intensity IDE has been shown to yield more significant benefits compared to lower-intensity interventions. However, increased intensity may lead to patient non-compliance or non-adherence during the intervention. The adherence rate has been reported to range from 71 to 91% [71], though some studies suggest that it could be lower, potentially impacting the intervention’s outcomes. For instance, Greenwood et al. (2021) observed a 47% completion rate of IDE, with only 18% adherence to exercise instructions [31]. This reduced compliance was likely due to fatigue and other side effects experienced during dialysis. Furthermore, compliance varies from patient to patient, and the intensity of IDE may also differ according to each individual’s capacity to tolerate the intervention. Despite this, some cases report high adherence rates, such as a German study documenting successful participation in an IDE program over 5 years [71]. The reasons behind such discrepancies remain unclear, but determining a balance between exercise intensity and patient adherence is essential for achieving good results from exercise programs.

Additionally, nutritional status is undoubtedly a factor that needs consideration regarding the effects of exercise. A study from Takahashi et al. [74] showed malnutrition’s negative impact on IDE in HD patients. This multicenter cohort study of 805 patients with reduced mobility found that IDE improved isometric knee extension strength (IKES), 10 m walking speed, and Short Physical Performance Battery scores in non-malnutrition, gentle malnutrition, and mild malnutrition groups but not in the severe malnutrition group [74]. The RCT by Geovana Martin-Alemañystudy et al. [46] found that oral nutritional supplementation improved muscle mass in HD patients, regardless of exercise intervention, and a synergistic effect emerged when nutritional supplementation was combined with IDE [46]. Nevertheless, controversial results exist. Noguchi et al. [75] demonstrated that nutritional status (serum albumin ≥ 3.6 g/dL versus < 3.6 g/dL) did not affect the effects of 12 weeks of IDE since both groups improved muscle strength after the IDE [75].

Additionally, we noticed that the age of patients may also be a factor influencing the effectiveness of exercise. A multicenter cohort study incorporating 1176 patients, grouped by ages of 40–59, 60–69, 70–79, and 80–89 years, concluded that the 10 min walking test and geriatric nutritional risk index were not improved in group 80-89 compared to other age groups; IKES in the age group 70-79 were not enhanced compared to the control group [76]. Further study might be warranted for evaluating the influence of age on exercise’s effects in HD patients. Furthermore, an issue worthy of further evaluation is demographic and cultural diversity since they can significantly influence the outcomes of studies evaluating the effects of exercise on HD patients. For example, the availability of resources (e.g., HD and IDE) and support from healthcare providers differ, impacting the implementation and success of exercise interventions [77]. Additionally, the different cultures associated with varied exercise perceptions significantly affect patients’ willingness to engage in exercise programs [78]. Understanding these cultural and contextual factors is crucial for designing effective and culturally sensitive exercise programs for HD patients.

### 6.3. Recommendations and Further Research Directions

A multidisciplinary approach is recommended to integrate IDE and HBE into routine HD care successfully. Collaboration between nephrologists, dialysis staff, and exercise specialists is crucial for developing tailored exercise programs considering patients’ health status and preferences. Additionally, implementing educational programs for healthcare providers and patients can increase awareness and adherence to IDE regimens, fostering a culture of proactive health management within dialysis [79]. Continuous monitoring and evaluation of the IDE programs’ effectiveness will contribute to refining protocols and optimizing outcomes over time [31]. Likewise, it is also crucial to conduct large-scale trials exploring the long-term effects on major clinical outcomes, including cardiovascular outcomes, improvement in physical performance, HRQoL, and the cost-effectiveness of IDE and HBE [70]. This information is essential for healthcare systems to justify resource allocation and support customized interventions.

## 7. Conclusions

In conclusion, the existing literature strongly supports the similar benefits of IDE and HBE in improving the health of HD patients. IDE and HBE enhance physical function, cardiopulmonary capacity, HRQoL, cognitive functions, and mental well-being. Some studies even suggest an indirect link between IDE and survival rates. Integrating these exercises into routine care allows healthcare providers to enhance outcomes for HD patients. Further research is needed to optimize exercise protocols and explore long-term effects and cost-effectiveness.

## Figures and Tables

**Table 1:** Summary of various IDE and HBE modalities.

Types of Exercise	Purpose	Implementation	Intensity	Duration and Frequency	
				IDE	HBE
Aerobic Exercise	To increase breathing and heart rate and maintain physical functions.	Walking, cycling, ergometer, or treadmill jogging.	Low-to-moderate intensity	At least 30 min/time, 2–3 times/week [9,10,11].	Up to 50 min/time, 2–3 times/week [20], or home-walking for 15–45 min daily [17].
Resistance Exercise	To increase muscle endurance and strength.	Body weight, weight machines, elastic bands, dumbbells, or repetition sets.	Low-to-moderate intensity	At least 2 times/week [10].	At least 60–90 min/time, 3 times/week, for 4–6 months [21,22].
Combined Aerobic-Resistance exercise	To increase breathing, heart rate, muscle endurance, muscle strength, and maintain physical functions.	Aerobic exercise with a cycling ergometer and resistance exercise with resistance bands, ankle weights, or dumbbells [23].	Moderate intensity	3 times/week for 6 weeks [23].	20 min to 2 h/time, 2–3 times/week, for 12 weeks [24,25].
Stretching Exercise	To enhance flexibility and reduce muscle cramps.	Implemented through simple flexibility and muscular extension exercises.	Very low intensity	At least 15 min/time, 3 times/week, for 2 months [26].	At least 10 min/time, 3 times/week, for 6 months [27].

Abbreviations: HBE, home-based exercise; IDE, intradialytic exercise.

**Table 2:** Merits of IDE and HBE.

Merits	IDE	HBE
Improving physical function	- Aerobic IDE improves lower extremity strength, 6MWT, STS, and HGS [35,36,37,38]. - Resistance IDE enhances lean leg muscle mass [39] and improves 6MWT, STS30, and HGS [38,40]. - Combined aerobic-resistance IDE improves lower extremity strength [35]. - Functional electrostimulation improves 6MWT [38]. - Inspiratory muscle training improves 6MWT [38].	- Aerobic HBE with gradually intensified low intensity improves 6MWT and STS5 [17]
Improving cardiopulmonary capacity	- Aerobic IDE improves VO_2peak_ [38,41]. - Combined aerobic-resistance IDE has benefited VO_2peak_, forced expiratory volume in the first second, forced vital capacity, peak expiratory flow, maximal inspiratory pressure [42], and cardiac autonomic nervous system activity [23,38].	- Combined aerobic-resistance HBE improves VO_2peak_ and cardiac autonomic nervous system activity [21]. - (Non-specified) HBE improves VO_2peak_ [30,32].
Improving HRQoL	- Aerobic IDE improves PCS and MCS of HRQoL [8,29,35]. - Resistance IDE improves PCS [43]. - Combined aerobic-resistance HBE improves PCS of HRQoL [8,29,35].	- Aerobic HBE with gradually intensified low intensity improves HRQoL [17].
Improving cognitive functions	- (Non-specified) Both IDE and HBE improve the cognitive condition (in those exercises for more than 16 weeks and lasts more than 30 min) [44].	- Aerobic HBE with gradually intensified low intensity improves cognitive function score [17]. - (Non-specified) Both IDE and HBE improve cognitive condition (in those exercises for more than 16 weeks and lasts more than 30 min) [44].
Improving patient survival	- Combined aerobic-resistance-stretching IDE for 6 months is associated with a lower mortality rate in the 12-month follow-up period [7].	N/A

Abbreviation: 6MWT, 6-Minute Walk Test; HBE, Home-based exercise; HGS, Hand grip strength; HRQoL, Health-related quality of life; IDE, Intradialytic exercise; MCS, Mental component summary; N/A, Not applicable; PCS, Physical component summary; STS, Sit-to-stand; STS5, 5-times sit-to-stand; STS30, 30 s sit-to-stand; VO_2peak_, Peak oxygen uptake.

## Data Availability

Not applicable.
